# Association of Phylogenomic Relatedness among *Neisseria gonorrhoeae* Strains with Antimicrobial Resistance, Austria, 2016–2020

**DOI:** 10.3201/eid2808.220071

**Published:** 2022-08

**Authors:** Justine Schaeffer, Kathrin Lippert, Sonja Pleininger, Anna Stöger, Petra Hasenberger, Silke Stadlbauer, Florian Heger, Angelika Eigentler, Alexandra Geusau, Alexander Indra, Franz Allerberger, Werner Ruppitsch

**Affiliations:** European Centre for Disease Prevention and Control, Stockholm, Sweden (J. Schaeffer);; Austrian Agency for Health and Food Safety, Vienna, Austria (J. Schaeffer, K. Lippert, S. Pleininger, A. Stöger, P. Hasenberger, S. Stadlbauer, F. Heger, A. Indra, F. Allerberger, W. Ruppitsch);; MB-LAB Clinical Microbiology Laboratory, Innsbruck, Austria (A. Eigentler);; Medical University of Vienna, Vienna (A. Geusau);; Paracelsus Medical University of Salzburg, Salzburg, Austria (A. Indra);; University of Natural Resources and Life Sciences, Vienna (W. Ruppitsch)

**Keywords:** Neisseria gonorrhoeae, antimicrobial resistance, whole-genome sequencing, core genome mutlilocus sequence typing, MLST, extended-spectrum cephalosporins, resistance genes, Austria, bacteria, sexually transmitted infections

## Abstract

We investigated genomic determinants of antimicrobial resistance in 1,318 *Neisseria gonorrhoeae* strains isolated in Austria during 2016–2020. Sequence type (ST) 9363 and ST11422 isolates had high rates of azithromycin resistance, and ST7363 isolates correlated with cephalosporin resistance. These results underline the benefit of genomic surveillance for antimicrobial resistance monitoring.

Gonorrhea, a sexually transmissible infection (STI) caused by *Neisseria gonorrhoeae*, is the second most common bacterial STI (*1*). Most gonorrhea cases are mild, but serious complications can occur. Gonorrhea is treated with antibiotics, and the recommended treatment is dual extended-spectrum cephalosporin (ESC)/azithromycin therapy or ceftriaxone monotherapy (*2*).

One of the main characteristics of *N. gonorrhoeae* is the plasticity of its genome, favoring the acquisition and dispersion of antimicrobial resistance (AMR). AMR is an increasing issue for gonorrhea treatment, and untreatable gonorrhea represents an imminent global health threat (*3*).

Whole-genome sequencing (WGS) provides high-resolution data that can support AMR surveillance. We combined phenotypic AMR testing with WGS to investigate 1,318 *N.* *gonorrhoeae* strains isolated in Austria during 2016–2020 and identify genetic risk factors associated with AMR.

## The Study

This study encompassed 1,318 *N. gonorrhoeae* isolates collected in Austria during 2016–2020; isolates were available at the National Reference Centre for Gonococci. We tested all isolates for phenotypic resistance to azithromycin, cefixime, ceftriaxone, ciprofloxacin, tetracycline, and benzylpenicillin, as well as production of β-lactamase (i.e., cefinase positive) (Appendix). We followed European Committee on Antimicrobial Susceptibility Testing guidelines (*4*) to determine MIC thresholds used in this study.

We performed genomic DNA isolation, WGS, assembly, and contig filtering as described previously (5) (Appendix). We deposited raw reads in the National Center for Biotechnology Information Sequence Read Archive (project no. PRJNA771206). We obtained sequences types (STs) from WGS data by using the PubMLST schemes (*6**,**7*). We generated a local *N. gonorrhoeae* core-genome multilocus sequence typing (cgMLST) scheme with SeqSphere+ target definer tool version 6.0.0 (Ridom, ttps://www.ridom.de) (*5*) (Appendix). We investigated AMR genes by using allele libraries based on PathogenWatch in TOML format version 0.0.14 (*8*).

We performed time series analysis, linear regression, univariate analysis, multivariate analysis (logistic regression), and data visualization by using R version 4.0.4 (Appendix). We defined statistical significance as p<0.05. We computed neighbor-joining trees in SeqSphere+ by using the number of cgMLST allelic differences and exported the trees into R.

**Figure 1:**
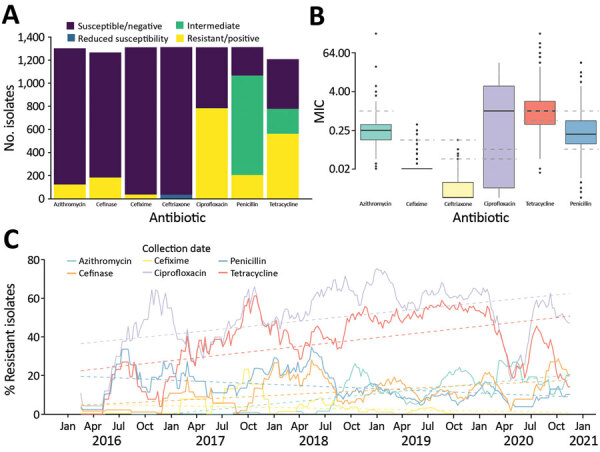
Antimicrobial resistance in 1,318 *Neisseria gonorrhoeae* isolates, Austria, 2016–2020. A) Number of isolates classified as susceptible, intermediate, or resistant. For ceftriaxone, isolates with reduced susceptibility are indicated in blue. For cefinase, β-lactamase producing isolates are indicated as positive (yellow). B) Boxplots of MIC obtained by Etest. Dashed lines indicate the thresholds used to classify the isolates as susceptible, intermediate, or resistant for ciprofloxacin, tetracycline, and penicillin, as susceptible or resistant for azithromycin, cefixime, and as susceptible, reduced susceptibility, or resistant for ceftriaxone. Horizontal lines within boxes indicate median, box tops and bottoms indicate quartiles 1 and 3, and dots indicate potential outliers. C) Evolution of the frequency of resistant isolates over time. Plain lines indicate the 13-week moving average of the percentage of isolates classified as resistant. Trends over time (obtained by linear regression) are represented by the dashed lines.

We classified isolates according to AMR (Figure 1, panel A; Table) and determined MIC distributions (Figure 1, panel B). We observed high levels of resistance to ciprofloxacin (60%) and tetracycline (46%) (Figure 1, panel A), which increased 5% per year for ciprofloxacin (p<0.0001) and 6% per year for tetracycline (p<0.0001). The percentage of penicillin-resistant isolates was 16% and decreased over the study period (2% per year; p<0.0001) (Figure 1, panel C); 14% of isolates were cefinase-positive, which increased by 2.7% per year (p<0.0001).

**Table:** Antimicrobial resistance classification and mean MIC of 1,318 *Neisseria gonorrhoeae* isolates, Austria, 2016–2020

Antibiotic	Antimicrobial resistance	No. isolates	Total no. isolates*	Frequency, %
Azithromycin	Susceptible (<1)	1,180	1,302	90.6
	Resistant (>1)	122	1,302	9.4
	MIC, µg/mL	0.8432 (0.2937–1.3927)		
Cefixime	Susceptible (<0.125)	1,276	1,311	97.3
	Resistant (>0.125)	35	1,311	2.7
	MIC, µg/mL	0.0289 (0.0266–0.0311)		
Ceftriaxone	Susceptible (<0.032)	1,279	1,312	97.5
	Reduced Sensitivity (>0.032)	33	1,312	2.5
	Resistant (>0.125)	0	1,312	
	MIC, µg/mL	0.007 (0.0064–0.0076)		
Ciprofloxacin	Susceptible (<0.032)	528	1,311	40.3
	Intermediate	1	1,311	0.1
	Resistant (>0.064)	782	1,311	59.6
	MIC, µg/mL	6.4455 (5.8446–7.0463)		
Tetracycline	Susceptible (<0.5)	431	1,208	35.7
	Intermediate	215	1,208	17.8
	Resistant (>1)	562	1,208	46.5
	MIC, µg/mL	7.0349 (5.9602–8.1096)		
Penicillin	Susceptible (<0.064)	246	1,312	18.8
	Intermediate	861	1,312	65.6
	Resistant (>1)	205	1,312	15.6
	MIC, µg/mL	2.2397 (1.8598–2.6196)		
Cefinase	Negative	1,083	1,266	85.5
	Positive	183	1,266	14.5
All		1,318		100

*Total number of isolates for which variable data were available.

We detected azithromycin resistance in 9% of the isolates, which increased by 5% per year (p<0.0001) (Figure 1). Two isolates from 2020 exhibited high levels of azithromycin resistance (MIC >256 µg/mL) but no other AMR. Resistance to ESC was rare; only 3% of isolates were resistant to cefixime, none were resistant to ceftriaxone, and 2.5% had reduced susceptibility to ceftriaxone (MIC >0.032 µg/mL). Cefixime resistance decreased by 0.9% per year (p<0.0001). Among cefixime-resistant isolates, 23/35 were resistant to ciprofloxacin and penicillin, qualifying as multidrug resistant.

**Figure 2:**
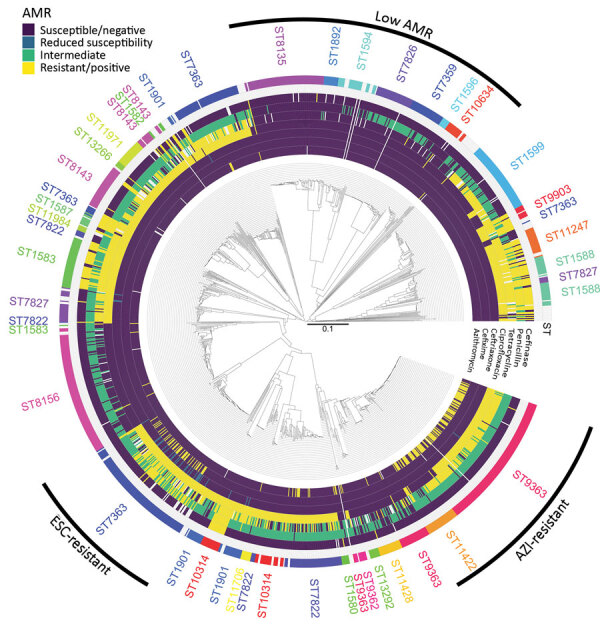
Correlation between population structure and antimicrobial resistance in *Neisseria gonorrhoeae* isolates, Austria, 2016–2020. Dendrogram was computed from the distance matrix of the core-genome multilocus sequence typing analysis (N = 1,304). Rims indicate the isolate classification as susceptible, intermediate, or resistant. For ceftriaxone, isolates with reduced susceptibility are indicated in blue. For cefinase, β-lactamase producing isolates are indicated as positive (yellow). The outer rim indicates sequence types corresponding to >2 consecutive isolates. Three branches with specific antimicrobial resistance patterns are indicated. AMR, antimicrobial resistance; AZI, azithromycin; ESC, extended-spectrum cephalosporin.

The isolates belonged to 119 different STs in mutlilocus sequence typing, including 23 newly defined (STs 15803–15825). The most prevalent STs were ST7363 (170 isolates), ST9363 (151 isolates), and ST8156 (113 isolates), which comprised 33% of the isolates. We identified 215 NG-MAST types for 873/1,318 isolates; the most prevalent STs were 12302 (73 isolates), 5441 (59 isolates), and 387 (50 isolates). cgMLST showed a branch including isolates with no or little AMR (Figure 2). We found no clear correlation with the cgMLST classification for penicillin, cefinase, tetracycline, and ciprofloxacin resistance. All cefixime-resistant isolates belonged to a single branch of ST7363 isolates, which also contained 24/32 isolates with reduced susceptibility to ceftriaxone. This branch had above average rates of ciprofloxacin, tetracycline, and penicillin resistance. A branch containing ST9363 and ST11422 isolates had a high rate of azithromycin resistance.

We searched isolate sequences for genes and point mutations associated with AMR (Appendix Table 3). For ciprofloxacin resistance, *gyrA* D95 substitutions were the main risk factor (adjusted odds ratio [aOR] 7.56 [95% CI 2.33–33.1]) and explained >99% of ciprofloxacin resistance. Tetracycline resistance was strongly associated with *tetM* carriage (aOR 157 [95% CI 48–965]), which we found in 33% of tetracycline-resistant isolates. For β-lactams, the main risk factor was *bla_TEM_* carriage (aOR 67.9 [95% CI 35.2–139] for penicillin and aOR 234 [95% CI 93.3–683] for cefinase). Mutations in *penA* were also associated with cefinase positivity (aOR 35.6 [95% CI 14–97.4]).

We found mutations in the *macAB* promoter or mosaic *mtr* genes in 138/149 azithromycin-resistant isolates (93%). All cefixime-resistant isolates carried *penA* G545S substitution. The major risk factor for reduced susceptibility to ceftriaxone was *penA* A501T/V (aOR 73.9 [95% CI 6.9–3,170]).

## Conclusions

This study combined phenotypic AMR and genomic data to analyze *N. gonorrhoeae* strains isolated in Austria during 2016–2020. We used a convenience sample (National Reference Centre for Gonococci collection) and results should be interpreted in light of this limitation. The percentage of *N. gonorrhoeae* strains resistant to azithromycin, ciprofloxacin, and tetracycline, or producing β-lactamase was increasing during the study period. The rate of azithromycin resistance rate was >13% during 2019–2020, which was high considering that an azithromycin/cefixime combination is a standard treatment for gonorrhea (*2*). We found no ceftriaxone-resistant isolates, and cefixime resistance rate was low.

We performed isolate typing by using multilocus sequence typing, *N. gonorrhoeae* multiantigen sequence typing (NG-MAST), and cgMLST. Only 37 isolates belonged to ST1901, which was predominant in isolates from Austria in a European study in 2013, highlighting the fast diversification of *N. gonorrhoeae* (*9*). The most common NG-MAST type was 12302; all isolates belonged to ST9363 and 71% were resistant to azithromycin. NG-MAST type 12302 and ST9363 have been associated with azithromycin resistance in other studies (*10*,*11*). cgMLST classification highlighted 3 branches with specific AMR patterns: 1 with low rates of AMR, 1 including azithromycin-resistant isolates, and 1 including ESC-resistant isolates. Previous studies comparing AMR and phylogenomic distributions in different countries showed either that azithromycin/ESC resistance emerged repeatedly in different networks or that their spread was largely clonal (*12*,*13*). In Austria, azithromycin and ESC resistance clustering was in favor of single introductions. The use of cgMLST among available classification methods has limitations (i.e., no counting of mutations within 1 gene, exclusion of intergenic regions, and resolution) but also advantages (i.e., no correction of recombination events necessary and one scheme fitting all isolates). This tool corresponds to the need for surveillance, where its lower resolution does not have a major effect.

We used our WGS data to search for genetic determinants of AMR (*8*,*14*). Ciprofloxacin resistance matched well with *gyrA* mutations (*9*,*12*). Tetracycline resistance correlated with *tetM*, and penicillin resistance correlated *bla_TEM_*. Mutations in *penA* and *mtrR* were associated with ESC resistance. Neither substitution C1192U in *16S rDNA* nor *rpsE* V25 mutations, associated with spectinomycin resistance, were found, suggesting a low prevalence of spectinomycin resistance.

Our study provides an overview of the *N. gonorrhoeae* strains circulating in Austria and their evolution over the past 5 years, both at the phenotypic and genomic level. It also underlines the benefits of genomic surveillance of *N. gonorrhoeae*, which can support epidemiologic investigations and provide information on specific genes and alleles thought to confer AMR (*14*).

Appendix Additional information about association of phylogenomic relatedness between *Neisseria gonorrhoeae* strains with antimicrobial resistance, Austria, 2016–2020.
