# Supplementary material for: Association of Phylogenomic Relatedness among Neisseria gonorrhoeae Strains with Antimicrobial Resistance, Austria, 2016–2020
Source: Emerg Infect Dis. 2022 Aug;28(8):1694–8. doi: 10.3201/eid2808.220071 (PMC9328923; doi:10.3201/eid2808.220071)
Supplement: Appendix — Additional information about association of phylogenomic relatedness between Neisseria gonorrhoeae strains with antimicrobial resistance, Austria, 2016–2020. [file 22-0071-Techapp-s1.pdf]

Article DOI: https://doi.org/10.3201/eid2808.220071

Association of Phylogenomic Relatedness between *Neisseria gonorrhoeae* Strains with Antimicrobial Resistance, Austria, 2016–2020

Appendix

Methods

Whole-Genome Sequencing

Genomic DNA isolation, WGS, assembly and contig filtering were performed as described previously (*1*). High-molecular-weight DNA was isolated from cultures using the MagAttract HMW DNA Kit (QIAGEN, Hilden, Germany), following the manufacturer's protocol for Gram-negative bacteria. Ready-to-sequence libraries were obtained with NexteraXT kit following the manufacturer's protocol (Illumina, CA, United States). Paired-end sequencing (2 × 300 bp) was performed on a MiSeq instrument as recommended by the manufacturer (Illumina). Raw reads were de novo assembled into a draft genome using SPAdes (version 3.11.1) (*2*). Contigs were filtered for a minimum coverage of 5 and minimum length of 200 bp. Sequencing quality was checked with FastQC. Sequencing generated 106,428 to 2,927,502 reads, a coverage of 12- to 272-fold (mean 76, 95% confidence interval [74.3–77.6]), a mean N50 of 38,513 (95% confidence interval [174–153,250]) and a mean contig length of 8,395 (95% confidence interval [208–23,184]).

Core-Genome MLST (cgMLST)

A local *N. gonorrhoeae* cgMLST scheme was generated with SeqSphere+ target definer tool (version 6.0.0, Ridom, Münster, Germany) (*3*). Strain MS11 was used as a seed genome (NCBI accession number NC_022240.1) and 47 complete *N. gonorrhoeae* genomes were used as query sequences (accession numbers NC_002946.2, NC_011035.1, NZ_CP012026.1, NZ_CP012027.1, NZ_CP012028.1, NZ_CP016015.1, NZ_CP016016.1, NZ_CP016017.1, ABZF00000000.1, ABZG00000000.1, ABZH00000000.1, ACIG00000000.1,

ADAA00000000.1, ABZJ00000000.2, ABZI00000000.1, ABZM00000000.1, ABZL00000000.1, ABZN00000000.1, ABZO00000000.1, ABZP00000000.1, ABZQ00000000.1, CQLK00000000.1, CQJM00000000.1, CQME00000000.1, CQJI00000000.1, CQIM00000000.1, CQHK00000000.1, CQLD00000000.1, CQNW00000000.1, CQKW00000000.1, CQJY00000000.1, CQIY00000000.1, CQJB00000000.1, CQKU00000000.1, CQOV00000000.1, CQIR00000000.1, CQJZ00000000.1, CQKM00000000.1, CQMI00000000.1, CQMT00000000.1, CQKB00000000.1, CQOT00000000.1, CQJD00000000.1, CHZN00000000.1, CFRU00000000.1, AKCG00000000.1, AKCH00000000.1), with default software parameters. A 1,524 loci cgMLST scheme and a 463 loci accessory target scheme were obtained, which were used in a previous publication (*4*).

Antimicrobial Resistance Genes Identifier, Adapted from PathogenWatch

Genotypic antibiotic resistance was investigated using allele libraries of *16S rDNA* (coding for 16S ribosomal RNA), *23S rDNA* (coding for 23S ribosomal RNA), *blaTEM*, *ereA*, *ereB*, *ermA*, *ermB*, *ermC*, *ermF*, *folP*, *gyrA*, *macAB promoter*, *mefA*, *mtrC*, *mtrR*, *mtrR promoter*, *mtr mosaic*, *norM promoter*, *parC*, *parE*, *penA*, *ponA1*, *porB1b*, *rplD*, *rplV*, *rpoB*, *rpoD*, *rpsE*, *rpsJ* and *tetM*, based on the library of PathogenWatch in TOML format (version 0.0.14) (*5*). Each allele library was implemented in SeqSphere+ (Ridom) and used to search assembled genomes. Alleles were matched if they reached 99% alignment to reference sequences. Alleles with >90% identity to reference sequences but no match were defined as "new allele" and aligned with reference sequences to identify mutations. All 1,318 study isolates were searched for genetic AMR using this tool.

Data Analysis

Statistical analysis was performed using R version 4.0.4. A positive outcome was defined as resistance to azithromycin, cefixime, ciprofloxacin, tetracycline, or penicillin, reduced susceptibility to ceftriaxone, or positivity for cefinase. For time series analysis, thirteen-weeks moving averages of collection dates were calculated (R packages ISOweek (*6*), zoo (*7*)). The percentage of resistant isolates (or with reduced susceptibility to ceftriaxone/positive for cefinase) over time was plotted, and trends were calculated by linear regression.

For risk factor identification, odds ratios (OR) were calculated for each outcome using univariate analysis (package epitools (*8*)). Multivariate analysis consisted in logistic regression including several explanatory variables (function glm and package broom (*9*)). Only genes or mutations reported to induce AMR to a given antibiotic by the PathogenWatch tool (*5*) were considered as potential explanatory variables. Explanatory variables were progressively included in the model until the lowest Akaike information criterion was reached. Adjusted odds ratio (aOR) were calculated

Data Visualization

Isolates were characterized by seven loci MLST scheme (*10*), NG-MAST (*11*) and by an in-house cgMLST scheme using SeqSphere+ (Ridom). Minimum spanning trees (MST) were computed using the number of cgMLST allelic differences between 1,304 isolates (14 were excluded due to <90% cgMLST good targets). Neighbor-joining tree (NJT) of the cgMLST analysis was exported from SeqSphere+ (Ridom, Münster, Germany) and loaded into R to compute dendrograms (packages ggplot2 (*12*), ggpubr (*13*), ape (*14*), ggtree (*15*)). Histograms and boxplots were created with R packages ggplot2 (*12*), viridis (*16*), RColorBrewer (*17*) and scales (*18*).

References

1. Lepuschitz S, Sorschag S, Springer B, Allerberger F, Ruppitsch W. Draft genome sequence of carbapenemase-producing *Serratia marcescens* isolated from a patient with chronic obstructive pulmonary disease. Genome Announc. 2017;5:e01288–17. PubMed https://doi.org/10.1128/genomeA.01288-17

2. Nurk S, Bankevich A, Antipov D, Gurevich AA, Korobeynikov A, Lapidus A, et al. Assembling single-cell genomes and mini-metagenomes from chimeric MDA products. J Comput Biol. 2013;20:714–37. PubMed https://doi.org/10.1089/cmb.2013.0084

3. Ruppitsch W, Pietzka A, Prior K, Bletz S, Fernandez HL, Allerberger F, et al. Defining and evaluating a core genome multilocus sequence typing scheme for whole-genome sequence-based typing of *Listeria monocytogenes.* J Clin Microbiol. 2015;53:2869–76. PubMed https://doi.org/10.1128/JCM.01193-15

4. Hirk S, Lepuschitz S, Cabal Rosel A, Huhulescu S, Blaschitz M, Stöger A, et al. Draft genome sequences of interpatient and intrapatient epidemiologically linked *Neisseria gonorrhoeae*

isolates. Genome Announc. 2018;6:e00319–18. PubMed https://doi.org/10.1128/genomeA.00319-18

5. Sánchez-Busó L, Yeats CA, Taylor B, Goater RJ, Underwood A, Abudahab K, et al. A community-driven resource for genomic epidemiology and antimicrobial resistance prediction of *Neisseria gonorrhoeae* at Pathogenwatch. Genome Med. 2021;13:61. PubMed https://doi.org/10.1186/s13073-021-00858-2

6. von Hatzfeld H. ISOweek: Week of the year and weekday according to ISO 8601. R package version 0.6–2. 2011 [cited 2022 Jan 8]. https://cran.r-project.org/web/packages/ISOweek/index.html

7. Zeileis A, Grothendieck G. zoo: S3 infrastructure for regular and irregular time series. [cited 2022 Jan 8]. J Stat Softw. 2005;14. https://doi.org/10.18637/jss.v014.i06

8. Aragon TJ. epitools: Epidemiology tools. R package version 0.5–10.1. 2020 [cited 2022 Jan 8]. https://cran.r-project.org/package=epitools

9. Robinson D, Hayes A, Couch S. broom: convert statistical objects into tidy tibbles. R package version 0.7.6. 2021 [cited 2022 Jan 8]. https://cran.r-project.org/web/packages/broom/index.html

10. Maiden MC, Bygraves JA, Feil E, Morelli G, Russell JE, Urwin R, et al. Multilocus sequence typing: a portable approach to the identification of clones within populations of pathogenic microorganisms. Proc Natl Acad Sci U S A. 1998;95:3140–5. PubMed https://doi.org/10.1073/pnas.95.6.3140

11. Martin IM, Ison CA, Aanensen DM, Fenton KA, Spratt BG. Rapid sequence-based identification of gonococcal transmission clusters in a large metropolitan area. J Infect Dis. 2004;189:1497–505. PubMed https://doi.org/10.1086/383047

12. Wickman H. ggplot2: elegant graphics for data analysis. 2016 [cited 2022 Jan 8]. https://cran.r-project.org/web/packages/ggplot2/index.html

13. Kassambara A. ggpubr: ‘ggplot2’ based publication ready plots. R package version 0.4.0. 2020 [cited 2022 Jan 8]. https://cran.r-project.org/web/packages/ggpubr/index.html

14. Paradis E, Schliep K. ape 5.0: an environment for modern phylogenetics and evolutionary analyses in R. Bioinformatics. 2019;35:526–8. PubMed https://doi.org/10.1093/bioinformatics/bty633

15. Yu G, Smith D, Zhu H, Guan Y, Tsan-Yuk Lam T. ggtree: an R package for visualization and annotation of phylogenetic trees with their covariates and other associated data. Methods Ecol Evol. 2017;8:28–36. https://doi.org/10.1111/2041-210X.12628

16. Garnier S. viridis: default color maps from ‘matplotlib’. R package version 0.5.1. 2018 [cited 2022 Jan 8]. https://cran.r-project.org/web/packages/viridis/index.html

17. Neuwirth E. RColorBrewer: ColorBrewer palettes. R package version 1.1–2. 2014 [cited 2022 Jan 8]. https://cran.r-project.org/web/packages/RColorBrewer/index.html

18. Wickham H. scales: scale functions for visualization. R package version 1.1.1. 2020 [cited 2022 Jan 8]. https://cran.r-project.org/web/packages/scales/index.html

Appendix Table 1. Measures of association between the different classes of antimicrobial resistance (N = 1,318)*

		Resistant			Susceptible			Univariate analysis	
AMR		tot	#	%	tot	#	%	OR [95%CI]	p.value
Azithromycin	Cefixime	121	0	0%	1180	35	3%	0 [0-NA]	0.06878
	Ceftriaxone	122	1	0.8%	1180	32	2.7%	0.296 [0.04–2.19]	0.3581
	Ciprofloxacin	122	88	72.1%	1179	688	58.4%	1.85 [1.22–2.79]	0.0035
	Tetracycline	121	86	71.1%	1077	472	43.8%	3.15 [2.09–4.75]	0
	**Penicillin**	122	2	1.6%	1180	202	17.1%	**0.081 [0.02–0.329]**	0
	**Cefinase**	121	1	0.8%	1135	181	15.9%	**0.044 [0.006–0.316]**	0
Cefixime	Azithromycin	35	0	0%	1266	121	9.6%	0 [0-NA]	0.06878
	Ceftriaxone	35	16	45.7%	1276	17	1.3%	**62.4 [27.5–142]**	0
	Ciprofloxacin	35	35	100%	1275	746	58.5%	Inf [NA-Inf]	0
	Tetracycline	27	19	70.4%	1180	542	45.9%	2.8 [1.21–6.44]	0.01761
	Penicillin	35	23	65.7%	1276	182	14.3%	11.5 [5.63–23.6]	0
	Cefinase	35	1	2.9%	1230	182	14.8%	0.169 [0.023–1.25]	0.04921
Ceftriaxone	Azithromycin	33	1	3%	1269	121	9.5%	0.296 [0.04–2.19]	0.3581
	**Cefixime**	33	16	48.5%	1278	19	1.5%	**62.4 [27.5–142]**	0
	Ciprofloxacin	33	32	97%	1278	750	58.7%	22.5 [3.07–165]	0
	Tetracycline	25	19	76%	1183	543	45.9%	3.73 [1.48–9.41]	0.00375
	Penicillin	33	22	66.7%	1279	183	14.3%	12 [5.71–25.1]	0
	Cefinase	29	2	6.9%	1237	181	14.6%	0.432 [0.102–1.83]	0.41838
Ciprofloxacin	Azithromycin	776	88	11.3%	525	34	6.5%	1.85 [1.22–2.79]	0.0035
	Cefixime	781	35	4.5%	529	0	0%	Inf [NA-Inf]	0
	Ceftriaxone	782	32	4.1%	529	1	0.2%	22.5 [3.07–165]	0
	Tetracycline	714	480	67.2%	494	82	16.6%	10.3 [7.76–13.7]	0
	**Penicillin**	782	200	25.6%	529	4	0.8%	**45.1 [16.6–122]**	0
	**Cefinase**	748	177	23.7%	517	5	1%	**31.7 [12.9–77.8]**	0
Tetracycline	Azithromycin	558	86	15.4%	640	35	5.5%	3.15 [2.09–4.75]	0
	Cefixime	561	19	3.4%	646	8	1.2%	2.8 [1.21–6.44]	0.01761
	Ceftriaxone	562	19	3.4%	646	6	0.9%	3.73 [1.48–9.41]	0.00375
	**Ciprofloxacin**	562	480	85.4%	646	234	36.2%	**10.3 [7.76–13.7]**	0
	Penicillin	562	141	25.1%	646	33	5.1%	6.22 [4.17–9.27]	0
	Cefinase	545	143	26.2%	617	19	3.1%	11.2 [6.83–18.4]	0
Penicillin	**Azithromycin**	204	2	1%	1098	120	10.9%	**0.081 [0.02–0.329]**	0
	Cefixime	205	23	11.2%	1106	12	1.1%	11.5 [5.63–23.6]	0
	Ceftriaxone	205	22	10.7%	1107	11	1%	12 [5.71–25.1]	0
	**Ciprofloxacin**	204	200	98%	1107	582	52.6%	**45.1 [16.6–122]**	0
	Tetracycline	174	141	81%	1034	421	40.7%	6.22 [4.17–9.27]	0
	**Cefinase**	194	150	77.3%	1072	33	3.1%	**107 [66.2–174]**	0
Cefinase	**Azithromycin**	182	1	0.5%	1074	120	11.2%	**0.044 [0.006–0.316]**	0
	Cefixime	183	1	0.5%	1082	34	3.1%	0.169 [0.023–1.25]	0.04921
	Ceftriaxone	183	2	1.1%	1083	27	2.5%	0.432 [0.102–1.83]	0.41838
	**Ciprofloxacin**	182	177	97.3%	1083	571	52.7%	**31.7 [12.9–77.8]**	0
	Tetracycline	162	143	88.3%	1000	402	40.2%	11.2 [6.83–18.4]	0
	**Penicillin**	183	150	82%	1083	44	4.1%	**107 [66.2–174]**	0

*A positive outcome was defined as resistance to azithromycin, cefixime, tetracycline and penicillin, reduced susceptibility to ceftriaxone and positivity for cefinase. For each variable, number of isolates (#), total number of isolates and frequency (%) are indicated for resistant (or with reduced susceptibility/positive) and susceptible (or negative) isolates. Odds ratio (OR) and 95% confidence interval were calculated by univariate analysis and association was tested with Fisher exact test.

Appendix Table 2. Genes and point mutations associated with antimicrobial resistance in *N. gonorrhoeae* isolates (N = 1,318). For each gene, number of isolates (#), total number of isolates for which the gene was found (tot) and frequency (%) are indicated

Gene	Variant	#	%
16S_rDNA	C1450	639	48.5%
	none	545	41.4%
	NA	134	10.2%
23S_rDNA	C2597	3	0.2%
	C2597.C265	1	0.1%
	C265	123	9.3%
	none	1124	85.3%
	NA	67	5.1%
blaTEM	not found	1185	89.9%
	found	133	10.1%
ereA	not found	1318	100%
	found	0	
ereB	not found	1318	100%
	found	0	
ermA	not found	1318	100%
	found	0	
ermB	not found	1318	100%
	found	0	
ermC	not found	1318	100%
	found	0	
ermF	not found	1314	99.7%
	found	4	0.3%
folP	R228	1081	0.82
	none	229	17.4%
	NA	8	0.6%
gyrA	D95	484	36.7%
	D95.S91	291	22.1%
	none	529	40.1%
	NA	14	1.1%
macAB_promotor	mut-10	129	9.8%
	none	1182	89.7%
	NA	7	0.5%
mefA	not found	1318	100%
	found	0	
mtr_mosaic	not found	1146	86.9%
	found	172	13.1%
mtrC	frameshift	23	1.7%
	none	1282	97.3%
	NA	13	1.0%
mtrR	found	1258	95.4%
	A39	337	25.6%
	A39.G45	48	3.6%
	frameshift	126	9.6%
	G45	160	12.1%
	none	637	48.3%
	NA	10	0.8%
mtrR_promoter	C187G	7	0.5%
	del-35	302	22.9%
	ins266A+ins253G	88	6.7%
	none	731	55.5%
	NA	190	14.4%
norM_promoter	ins211	62	4.7%
	ins211.ins250	124	9.4%
	ins250	5	0.4%
	none	1117	84.7%
	NA	10	0.8%
parC	D86N	284	21.5%
	E91G/Q	80	6.1%
	E91G/Q.S87I	12	0.9%
	E91G/Q.S87N	20	1.5%
	E91K.S87N	25	1.9%
	S87I	1	0.1%
	S87N	29	2.2%
	S87N.S88P	2	0.2%
	S87R	198	0.15
	S87R.S88P	103	7.8%
	S88P	21	1.6%

Gene	Variant	#	%
	none	529	40.1%
	NA	14	1.1%
penA	A501T/V	1	0.1%
	A501T/V.ins346D	63	4.8%
	A501T/V.ins346D.P551S/L	94	7.1%
	G545S.I312M+V316T.I312M+V316T	304	23.1%
	I312M+V316T	2	0.2%
	I312M+V316T.P551S/L	1	0.1%
	ins346D	706	53.6%
	ins346D.P551S/L	16	1.2%
	none	119	0.09
	NA	12	0.9%
ponA1	L421	561	42.6%
	none	738	0.56
	NA	19	1.4%
porB1b	A121	162	12.3%
	A121.G120	392	29.7%
	G120	33	2.5%
	none	579	43.9%
	NA	152	11.5%
rplD	G68	18	1.4%
	G70	40	0.03
	none	1252	0.95
	NA	8	0.6%
rpsE	D11	141	10.7%
	none	1170	88.8%
	NA	7	0.5%
rpsJ	V57	961	72.9%
	none	342	25.9%
	NA	15	1.1%
tetM	not found	1061	80.5%
	found	257	19.5%
All		1318	100%

Appendix Table 3. Genetic risk factors associated with resistant *N. gonorrhoeae* isolates (N = 1,318)*

	Patient data		Resistant			Susceptible			Univariate analysis		multivariate analysis	
Amr	Variable	Category	Tot	#	%	Tot	#	%	OR [95%CI]	p.value	OR [95%CI]	p.value
Azithromycin	23S_rDNA	C265T	115	3	2.6%	1122	120	10.7%	0.224 [0.07–0.715]	0.00294		
	macAB_promotor	mut-10	120	80	66.7%	1175	48	4.1%	**47 [29.1–75.7]**	**0**	**27.7 [1.3–231]**	**0.00566**
	mtrR_promoter	del-35	12	7	58.3%	1101	293	26.6%	3.86 [1.22–12.3]	0.02107	2.59 [0.594–11.2]	0.18732
	mtr_mosaic	found	122	103	84.4%	1180	68	5.8%	**88.7 [51.3–153]**	**0**		
	mtrR	A39T	120	11	9.2%	1172	370	31.6%	0.219 [0.116–0.412]	0		
		frameshift	120	1	0.8%	1172	121	10.3%	**0.073 [0.01–0.527]**	**0.00012**		
		G45D/S	120	3	2.5%	1172	204	17.4%	0.122 [0.038–0.387]	1.00E-06	1.54e-07 [NA-1.63e+37]	0.98996
Cefixime	mtrR_promoter	del-35	35	0	0%	1088	302	27.8%	0 [0-NA]	2.00E-05	3.95e-09 [2.43e-312–9.92e+39]	0.99335
	mtrR	A39T	35	0	0%	1266	383	30.3%	0 [0-NA]	6.00E-06	3.62e-09 [1.94e-281–1.05e+35]	0.99256
		G45D/S	35	29	82.9%	1266	179	14.1%	**29.4 [12–71.7]**	**0**	2.27 [0.875–6.71]	0.10868
	penA	G545S	35	35	100%	1264	268	21.2%	Inf [NA-Inf]	0	3.7e+08 [4.46e+25–1.06e+258]	0.98916
Ceftriaxone	penA	A501T/V	32	5	15.6%	1268	153	12.1%	1.35 [0.512–3.56]	0.57969	**73.9 [6.9–3.17e+03]**	**0.00421**
		G545S	32	26	81.2%	1268	277	21.8%	**15.5 [6.32–38]**	**0**	**16.2 [2.95–369]**	**0.01321**
		I312M+V316T	32	26	81.2%	1268	280	22.1%	**15.3 [6.23–37.5]**	**0**		
		ins346D	32	5	15.6%	1268	869	68.5%	**0.085 [0.033–0.222]**	**0**	**0.0723 [0.00231–2.18]**	**0.09253**
Ciprofloxacin	gyrA	D95N/G/A/Y	772	767	99.4%	525	5	1%	**1.6e+04 [4.6e+03–5.54e+04]**	**0**	**7.56e+03 [2.33e+03–3.31e+04]**	**8.66E-27**
		S91F/T	772	290	37.6%	525	1	0.2%	**315 [44.1–2.25e+03]**	**0**		
	norM_promoter	ins211A	775	161	20.8%	526	24	4.6%	5.49 [3.52–8.56]	0	8.11 [1.04–53.2]	0.03637
		ins250T	775	117	15.1%	526	11	2.1%	8.32 [4.44–15.6]	0		
	parC	D86N	774	252	32.6%	523	29	5.5%	8.22 [5.49–12.3]	0	4.5 [0.814–26.8]	0.09268
		E91G/Q	774	91	11.8%	523	22	4.2%	3.03 [1.88–4.9]	1.00E-06		
		S87N	774	75	9.7%	523	1	0.2%	**56 [7.76–404]**	**0**		
		S87R	774	299	38.6%	523	2	0.4%	**164 [40.6–662]**	**0**	**24.2 [2–463]**	**0.03343**
Tetracycline	mtrR_promoter	del-35	437	128	29.3%	584	151	25.9%	1.19 [0.9–1.57]	0.22835	5.3 [3.29–8.71]	1.74E+02
	mtrR	A39T	560	162	28.9%	639	190	29.7%	0.962 [0.75–1.23]	0.79937	0.535 [0.306–0.921]	0.02552
		G45D/S	560	110	19.6%	639	79	12.4%	1.73 [1.26–2.37]	0.00062	2.52 [1.53–4.2]	0.00033
	rpsJ	V57M	555	552	99.5%	639	323	50.5%	**180 [57.3–566]**	**0**	**113 [41.1–467]**	**3.18E-01**
	tetM	found	562	223	39.7%	646	3	0.5%	**141 [44.8–444]**	**0**	**345 [104–2.14e+03]**	**1.44E-01**
Penicillin	blaTEM	found	205	100	48.8%	1107	33	3%	**31 [19.9–48.2]**	**0**	**67.9 [35.2–139]**	**1.08E-19**
	mtrR_promoter	del-35	203	30	14.8%	920	272	29.6%	0.413 [0.273–0.624]	1.1e-05	0.257 [0.118–0.53]	0.00035
		ins266A+ins253G	203	20	9.9%	920	68	7.4%	1.37 [0.811–2.31]	0.24839	3.55 [1.42–8.45]	0.00501
	mtrR	A39T	204	47	23%	1098	336	30.6%	0.679 [0.478–0.964]	0.02974		
		frameshift	204	34	16.7%	1098	92	8.4%	2.19 [1.43–3.35]	0.00068		
		G45D/S	204	74	36.3%	1098	134	12.2%	4.09 [2.92–5.74]	0	2.29 [1.18–4.42]	0.01352
	penA	G545S	203	77	37.9%	1097	226	20.6%	2.35 [1.71–3.24]	0		
		I312M+V316T	203	77	37.9%	1097	229	20.9%	2.32 [1.68–3.19]	1.00E-06		
		P551S/L	203	9	4.4%	1097	102	9.3%	0.453 [0.225–0.91]	0.02004	0.368 [0.121–1.01]	0.06114
	ponA1	L421P	203	136	67%	1090	424	38.9%	3.19 [2.32–4.38]	0	4.5 [2.3–8.95]	1.28E+08
	porB1b	A121D/N/S/G/V	173	101	58.4%	988	451	45.6%	1.67 [1.2–2.32]	0.00222		
		G120K/N/D/Q/R	173	113	65.3%	988	311	31.5%	4.1 [2.92–5.76]	0	6.15 [3.16–12.3]	1.60E+07
Cefinase	blaTEM	found	183	118	64.5%	1083	13	1.2%	**149 [80–279]**	**0**	**234 [93.3–683]**	**2.73E-13**

Amr	Patient data Variable	Category	Resistant Tot	#	%	Susceptible Tot	#	%	Univariate analysis OR [95%CI]	p.value	multivariate analysis OR [95%CI]	p.value
	mtrR_promoter	del-35	180	25	13.9%	898	267	29.7%	0.381 [0.244–0.595]	6.00E-06	0.0892 [0.0248–0.277]	8.56E+09
		ins266A+ins253G	180	21	11.7%	898	64	7.1%	1.72 [1.02–2.9]	0.04805	5.77 [2.23–14.2]	0.00017
	mtrR	A39T	182	76	41.8%	1074	300	27.9%	1.85 [1.34–2.56]	0.00023		
		frameshift	182	35	19.2%	1074	88	8.2%	2.67 [1.74–4.09]	2.00E-05		
	penA	A501T/V	181	56	30.9%	1073	97	9%	4.51 [3.09–6.58]	0	**35.6 [14–97.4]**	**3.43E+01**
		G545S	181	29	16%	1073	253	23.6%	0.618 [0.406–0.942]	0.02655		
		I312M+V316T	181	29	16%	1073	255	23.8%	0.612 [0.402–0.933]	0.02117		
		ins346D	181	150	82.9%	1073	706	65.8%	2.52 [1.68–3.78]	3.00E-06	3.49 [1.66–7.57]	0.00116
		P551S/L	181	8	4.4%	1073	97	9%	0.465 [0.222–0.974]	0.04125	0.107 [0.0287–0.378]	0.00058
	porB1b	A121D/N/S/G/V	147	52	35.4%	973	472	48.5%	0.581 [0.405–0.833]	0.00334		
		G120K/N/D/Q/R	147	60	40.8%	973	336	34.5%	1.31 [0.917–1.86]	0.13973	4.56 [2.17–9.67]	6.61E+09

*A positive outcome was defined as resistance to azithromycin, cefixime, tetracycline and penicillin, reduced susceptibility to ceftriaxone and positivity for cefinase. For each variable, number of isolates (#), total number of isolates and frequency (%) are indicated for resistant (or with reduced susceptibility/positive) and susceptible (or negative) isolates. For univariate analysis, odds ratio (OR) and 95% confidence interval were calculated and association was tested with Fisher exact test. For multivariate analysis, variables with significant association in univariate analysis were included in a logistic regression model. Adjusted odds ratio (aOR), 95% confidence interval and p_{value} were calculated for the model with the lowest Akaike information criterion.
